# Immunohistochemical expression of MMP-14 and MMP-2, and MMP-2 activity during human ovarian follicular development

**DOI:** 10.1186/1477-7827-12-12

**Published:** 2014-01-31

**Authors:** Maria Caroline Vos, Anneke AM van der Wurff, Jessie TJ Last, Ella AM de Boed, Jesper MJ Smeenk, Toin H van Kuppevelt, Leon FAG Massuger

**Affiliations:** 1 Department of Obstetrics and Gynaecology, St. Elisabeth Hospital, PO Box 90151, 5000 LC Tilburg, the Netherlands; 2 Department of Pathology, St. Elisabeth Hospital, PO Box 90151, 5000 LC Tilburg, the Netherlands; 3 Department of Biochemistry, Nijmegen Centre for Molecular Life Sciences, Radboud university medical center, PO Box 9101, 6500 HB Nijmegen, the Netherlands; 4 Department of Obstetrics and Gynaecology, Radboud university medical center, PO Box 9101, 6500 HB Nijmegen, the Netherlands

**Keywords:** Female reproductive tract, Ovary, Menstrual cycle, Matrix metalloproteinases

## Abstract

**Background:**

The aim of this study was to investigate the presence of MMP-14 and MMP-2 during human ovarian follicular development using immunohistochemistry, and the activity of MMP-2 in follicular fluid using zymography.

**Methods:**

Ovarian tissue collected from the archives of the Department of Pathology was examined and medical records and histopathology were reviewed. Follicular fluids were collected at the IVF-department and analyzed using zymography.

**Results:**

MMP-14 and MMP-2 were increasingly found in the growing follicles and MMP-2 was highly expressed in the corpus luteum. Pro-MMP-2 was present in follicular fluid of IVF-patients.

**Conclusions:**

The presence of MMP-14 and MMP-2 during human ovarian follicular development from the primordial follicle to the tertiary follicle and corpus luteum is confirmed, as was indicated by earlier animal studies following stimulation with gonadotrophins.

## Background

Proteins of the matrix metalloproteinase (MMP) family are involved in degrading the extracellular matrix in normal physiological processes such as embryonic development, reproduction, and tissue remodeling, as well as in disease processes such as wound healing, arthritis and cancer [1]. Most MMP’s are secreted as inactive proproteins, which are activated when cleaved by extracellular proteinases. For this study, we focused on MMP-14 and MMP-2.

MMP-14 (former MT1-MMP) is a member of the membrane-type MMP (MT-MMP) subfamily [2]. These proteins are expressed at the cell surface rather than secreted, and contain a transmembrane domain. Apart from functioning as a gelatinase itself, MMP-14 also cleaves pro-MMP-2 (72 kD) into its active 66 kD form [2,3]. Holmbeck et al. described MMP-14 deficient mice, especially showing malfunctioning of the connective tissue of ligaments, tendons and joint capsules. MMP-14 was found to have a postnatal effect. The mice show no sexual maturation and age early with generalized fibrosis, loss of hair, joint contractures and reduced mobility [1,4].

Mice deficient in MMP-2 show normal development and reproduction, but have specific altered cellular functions spontaneously or on experimental challenge [1]. Although MMP’s seem to play an important role in ovulation, the role of MMP’s may be taken over by other proteins, as the studies with MMP-inhibitors in rodents on corpus luteum formation demonstrates [5].

Animal research in rodents, cattle and primates suggests a role for MMP-14 and MMP-2 in ovarian follicle-formation and follicle-rupture [6,7]. In normal follicle growth in humans, the follicle expands to 400 times its size in approximately two weeks before timely ovulation. This requires coordinated remodeling of the basement membrane of the follicle and the surrounding extra-cellular matrix. Gelatinase activity by MMP-2 is found in follicular fluid on the inside of the follicle and higher activity is found in PolyCystic Ovary Syndrome (PCOS) patients compared to women with normal ovulation [8]. MMP-2 also plays a role in corpus luteum formation in humans. However, the sizes of these immunohistochemical studies in humans are rather small with a total of 78 patients [9-12].

Little is known about the exact role of MMP-14 and MMP-2 in human follicular growth and development, as tissue is not readily available. Therefore, the role of MMP-14 and MMP-2 in ovarian physiology is difficult to investigate. Furthermore, it is impossible to test the same follicle while growing and after ovulation. Even animal research is hampered by sampling restrictions of follicles and the fact that sampling in developing follicles might induce MMP-activity due to the repeated trauma of the follicle.

By studying MMP-14 and MMP-2 with immunohistochemistry in normal ovaries, the presence of these proteins in follicles in various stages of development can be evaluated. In this study we present data on the expression of MMP-14 and MMP-2 in human follicular development, and zymographic activity of MMP-2 in follicular fluid.

## Methods

From the Database of the Clinical Pathological Laboratory patients were selected with the search term “normal ovary”. From these patients paraffin-embedded blocks that were stored at room temperature within the timeframe 2009–2010 were collected from the archives. Of 30 consecutive patients the medical records were studied. Only premenopausal patients, who were not using hormonal therapy (such as oral contraceptives and levonorgestrel containing IUD) were included. All patients had a regular cycle. Patients with ovarian malignancies and benign ovarian tumors which are known to have MMP-expression (e.g. endometriosis) were excluded. One ovary was normal and was removed because of a mucinous borderline tumor of the other ovary. Two patients had a teratoma, one patient had a serous cystadenoma, two patients had a mucinous cystadenoma, one patient had a simple cyst, one a pseudocyst and one a functional cyst. All 9 ovaries showed normal histopathology before and after revision, the ovaries with torsion showed no lysis or haemorrhagia. As well as histopathology, clinical patient data were reviewed.

Sections (3 μm) were deparaffinised in xylene and rehydrated in graded alcohol. Immunohistochemistry was done in one run with omitting the primary antibody as a negative control.

Each slide contained positive controls for MMP’s, consisting of placenta. Endogenous peroxidase was blocked with 3% H_2_O_2_ and Normal Goat Serum 5%. After each incubation step, slides were washed two times with Phosphate Buffered saline (PBS).

For MMP-14 we used a polyclonal antibody, anti-human MMP-14 rabbit IgG (Thermo Scientific), diluted 1:20 for 60 minutes at room temperature [2].

As primary antibody for MMP-2, a monoclonal antibody was used, anti-human MMP-2 mouse IgG (clone A-Gel vc2, Thermo Scientific), diluted 1:10 [13]. Incubation was overnight at 4° Celsius.

As secondary antibody for both proteins poly-HRP-GAM/R/R IgG (Immunologic, Duiven, the Netherlands) was used during 60 minutes at room temperature. Staining was done with diaminobenzidine for two times 5 minutes using the Power Vision protocol. Slides were counterstained with hematoxylin.

A scoring system was used in which the intensity of the scoring (-absent, +weak, ++moderate, +++strong staining) and the number of positive follicular cells (0 = 0%, 1 = 1-25%, 2 = 26-50%, 3 = 51-75%, 4 = 76-100% of cells) were incorporated [14]. Stromal staining was recorded, but not incorporated in the scoring system. Follicles in different phases of development were scored according to our scoring system.

Two investigators scored all slides (JL, MCV). If they disagreed, consensus was found by consulting the gynecopathologist (AvdW).

Of 9 consecutive IVF-ICSI patients at the Centre for Reproductive Medicine at St. Elisabeth Hospital, Tilburg, the Netherlands, follicular fluid was collected at the time of IVF puncture. Also, cyst fluid of a patient with a benign cyst was collected. Of these patients the following variables were collected: age, duration of infertility, indication for IVF-ICSI, number of oocytes and the result of treatment in terms of pregnancy.

Follicular fluid was collected in 50 ml tubes, of which the first tube was used for zymographic analysis. At the time of puncture the total amount of fluid per tube and the appearance of the follicular fluid, the number of oocytes in that tube and the total number of oocytes were described. Follicular fluid was frozen at -20° until time of analysis.

The protocol was reviewed by our Medical Ethical Committee that confirmed, that it did not need formal evaluation. IVF-ICSI patients were informed by a leaflet describing this investigation.

Zymography was performed according to [15]. In short, 10% SDS-PAGE gels containing 2 mg gelatin/ml were loaded with 10 μl follicular fluid in non-reducing sample buffer 1:1. After electrophoresis, gels were washed twice with 2.5% Triton X-100 (BDH Chemicals) for 15 min. After a final wash in 0.05 M Tris HCl, pH 8.0 containing 5 mMCaCl_2_ and 0.01% Triton X-100 gels were incubated in 0.05 M Tris HCl, pH 8.0 containing 5 mM CaCl_2,_ 0.01% Triton X-100 and 5 mM ZnCl_2_ at 37% for 4–72 h. After incubation gels were stained with 0.5% (w/v) Coomassie Brilliant Blue R250 in a mixture of methanol, acetic acid and MilliQ (5:1:4 resp.) After destaining in 5:1:4 methanol/acetic acid/MilliQ, zones of enzymatic activity appeared as clear white bands against a blue background.

Gels were incubated in the presence of 20 mM EDTA, an inhibitor of MMP’s, to indicate the specificity of the assay.

Because of the low number of patients for both immunohistochemistry as well as zymography formal statistical analysis was not performed.

## Results

We examined histopathological slides of 9 ovaries of patients, that were removed because of benign, functional or simple cysts, two of which underwent torsion. For histopathological characteristics of the ovaries see Table 1.

**Table 1:** Histopathological characteristics of ovaries used for immunohistochemical studies

**Age**	**Histopathological diagnosis**	**Treatment**	**Remarks**
32	Teratoma	Laparoscopic cystectomy	
55	Simple cyst	Salpingo-oophorectomy	
33	Serous cystadenoma	Cystectomy	
35	Pseudocyst	Salpingo-oophorectomy	
36	Functional cyst	Laparoscopic cystectomy	Torsion
34	Teratoma	Laparoscopic cystectomy	Torsion
38	Mucinous cystadenoma and Brenner tumour	Laparoscopic ovariectomy	
34	Mucinous cystadenoma	Cystectomy	
48	Normal ovary	Staging laparotomy	Mucinous borderline tumor other ovary

We found mainly cytoplasmatic staining for MMP-14 and mainly pericellular staining for MMP-2. The MMP-14 staining was more diffuse than the MMP-2 staining.

In Table 2 the main results of immunohistochemistry are summarized. Figures 1, 2, 3, 4, 5, 6, 7, 8, 9, 10, 11 and 12 show examples of relevant sections.

**Table 2:** IHC scores in ovarian follicular development

**Histopathological diagnosis**	**OSE**	**Primord. Follicle**	**PF**	**TF granulosa cells**	**TF theca cells**	**CL granulosa cells**	**CL theca cells**	**Atretic follicle**
Teratoma		17+/19*-		++/**	+/*			
Simple cyst	++/**							-/-
Serous cystadenoma	++/*					+++/*		
Pseudocyst	++/*	2+/9-	+/*					
Functional cyst (torsion)	+/*			++/**	+/*			
Teratoma (torsion)		4+/6-						
Mucinous cystadenoma and Brenner tumour	++/*	2+/1-						
Mucinous cystadenoma	+		/-					
Normal ovary	+/*	+/*						

IHC = ImmunoHistoChemistry, OSE = Ovarian Surface Epithelium, Primord. = Primordial, PF = Primary/Growing Follicle, TF = Tertiary Follicle, CL = Corpus Luteum, - = no MMP-2 or 14 expression, + = MMP-2 expression, * = MMP-14 expression, ++ = moderate MMP-2 expression, ** = moderate MMP-14 expression, +++ = strong MMP-2 expression, the number before the symbols -, + or * indicates the number of follicles found, if no number is indicated, the number is one.

**Figure 1:**
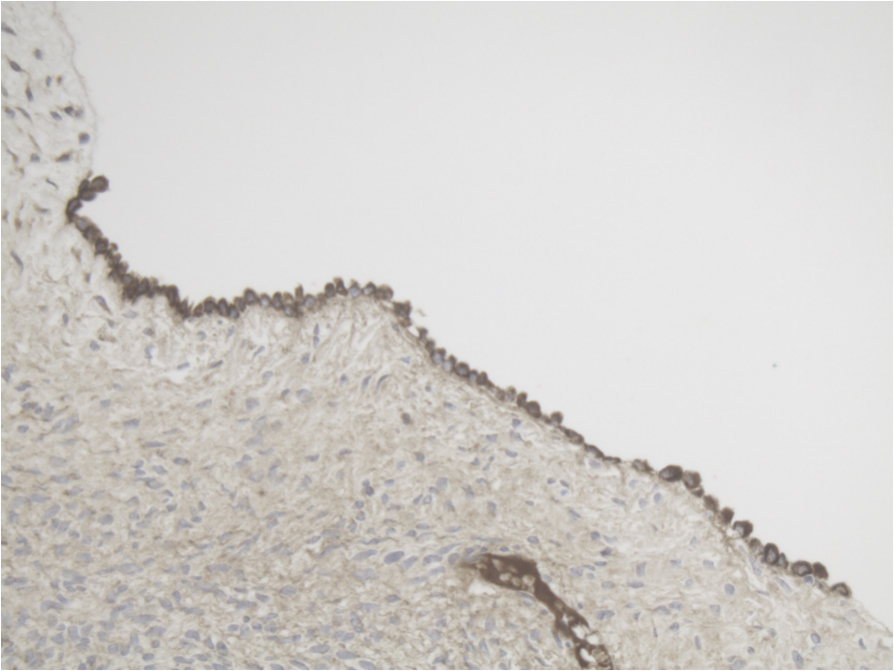
Representative slide of Ovarian surface epithelium for MMP-2 10×20 magnification showing high pericellular expression of MMP-2.

**Figure 2:**
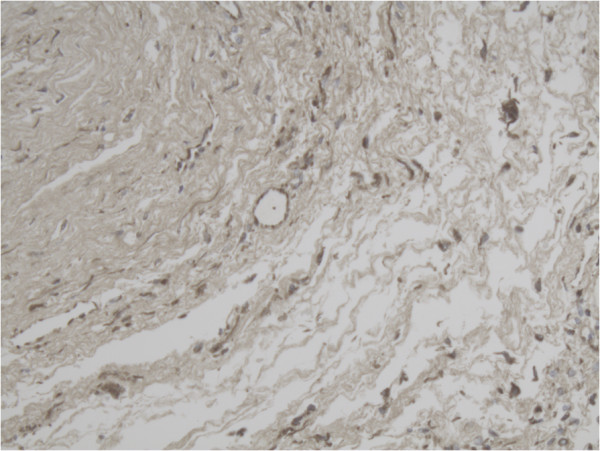
Representative slide of Primordial follicle for MMP-2 10×20 magnification showing MMP-2 expression in most cells.

**Figure 3:**
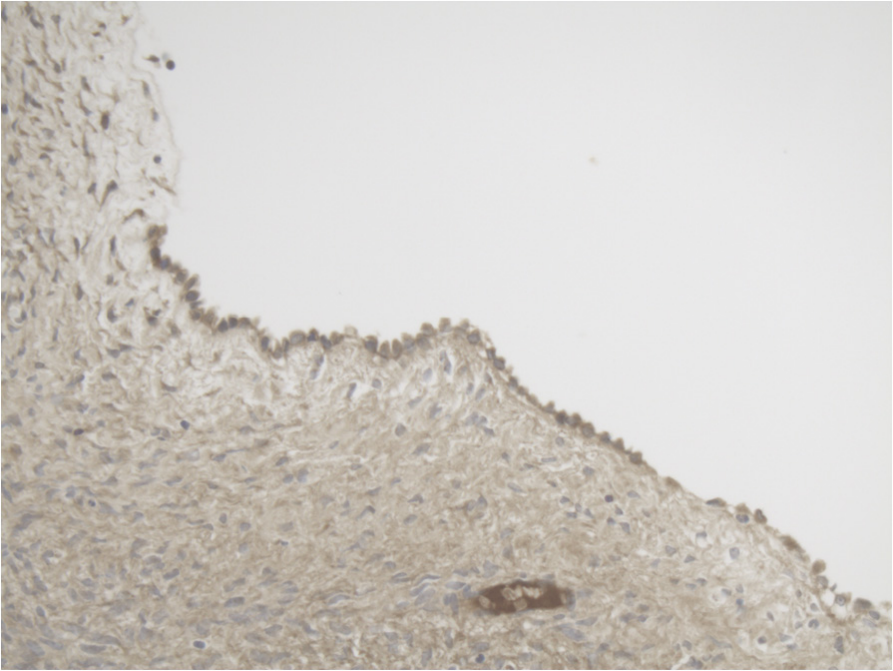
Representative slide of Ovarian surface epithelium for MMP-14 10×20 magnification showing diffuse cytoplasmatic expression of MMP-14.

**Figure 4:**
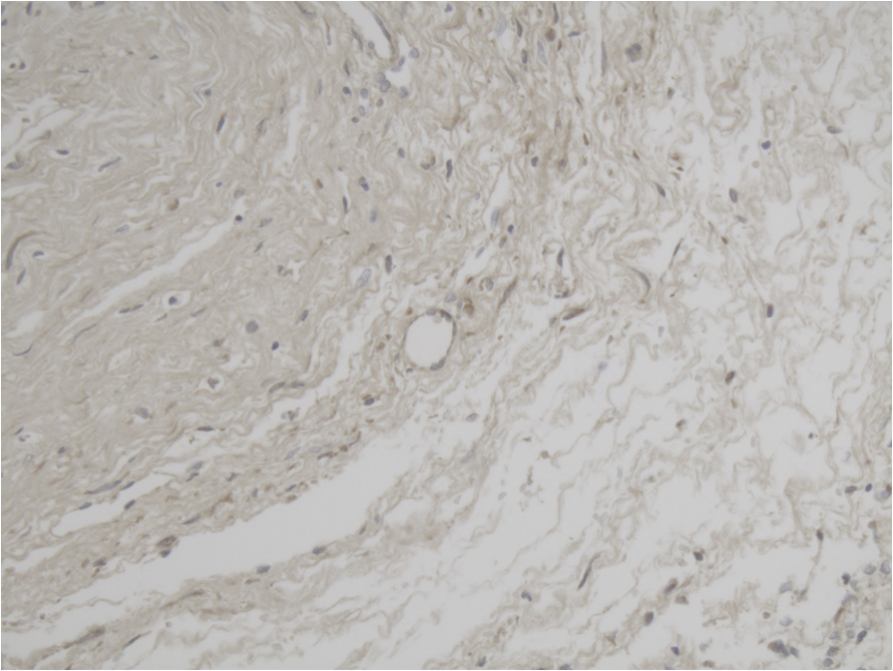
Representative slide of Primordial follicle for MMP-14 10×20 magnification showing MMP-14 expression in some cells and not in other cells.

**Figure 5:**
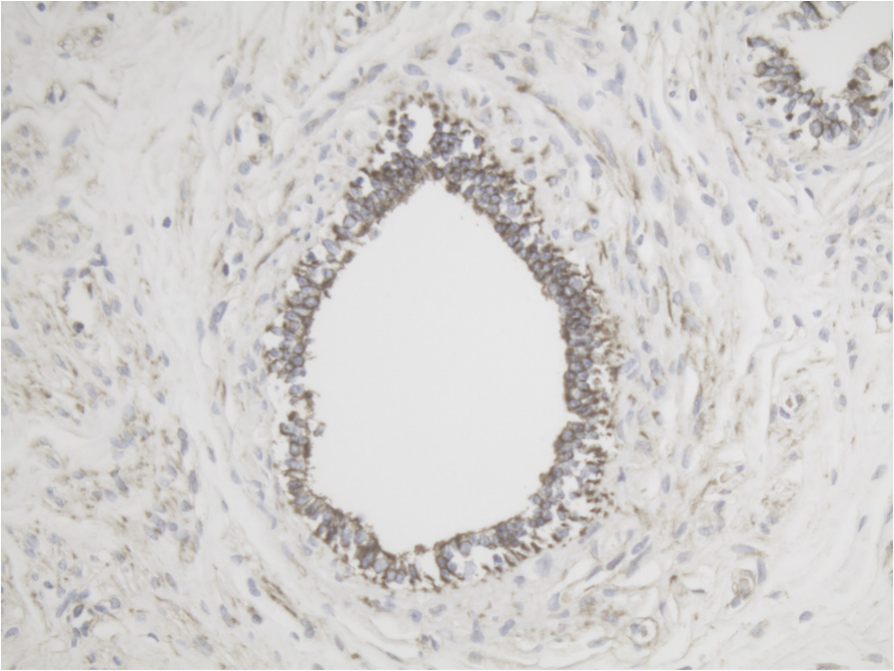
Representative slide of Primary follicle for MMP-2 10×20 magnification showing intense expression of MMP-2 in most cells.

**Figure 6:**
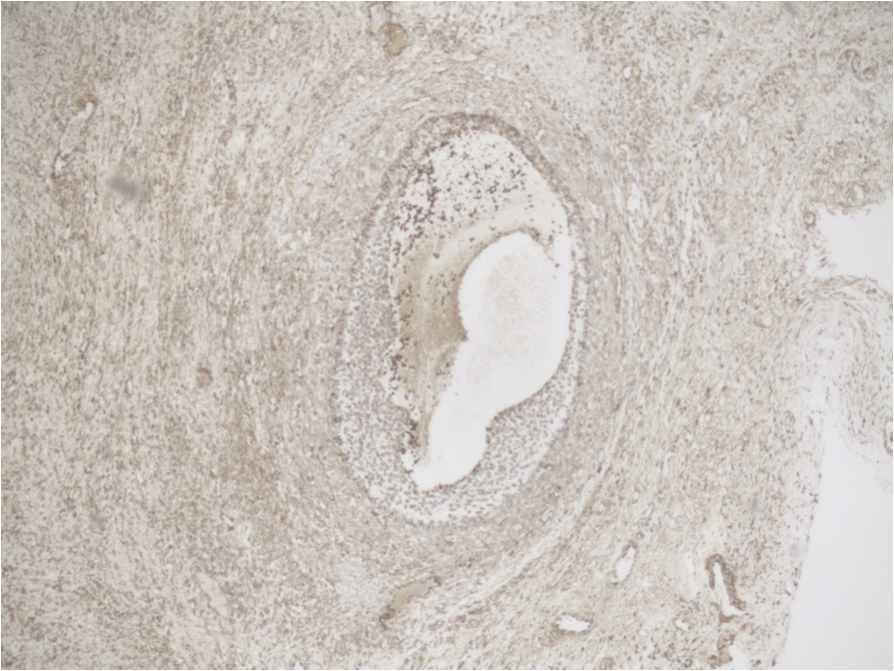
Representative slide of overview Tertiary follicle for MMP-2 5×10 magnification.

**Figure 7:**
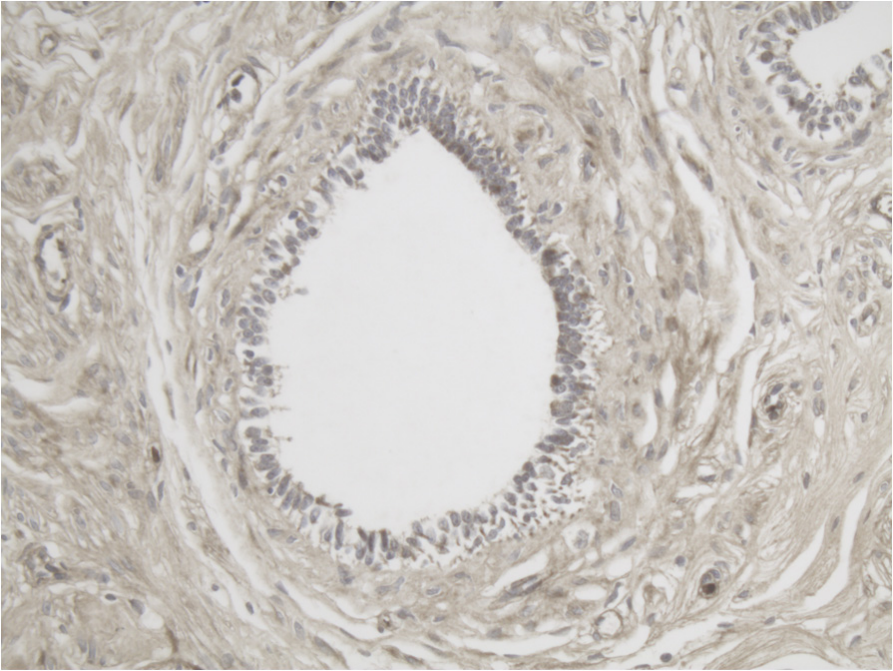
Representative slide of Primary follicle for MMP-14 10×20 magnification showing expression of MMP-14 in especially the inner layer of the follicle.

**Figure 8:**
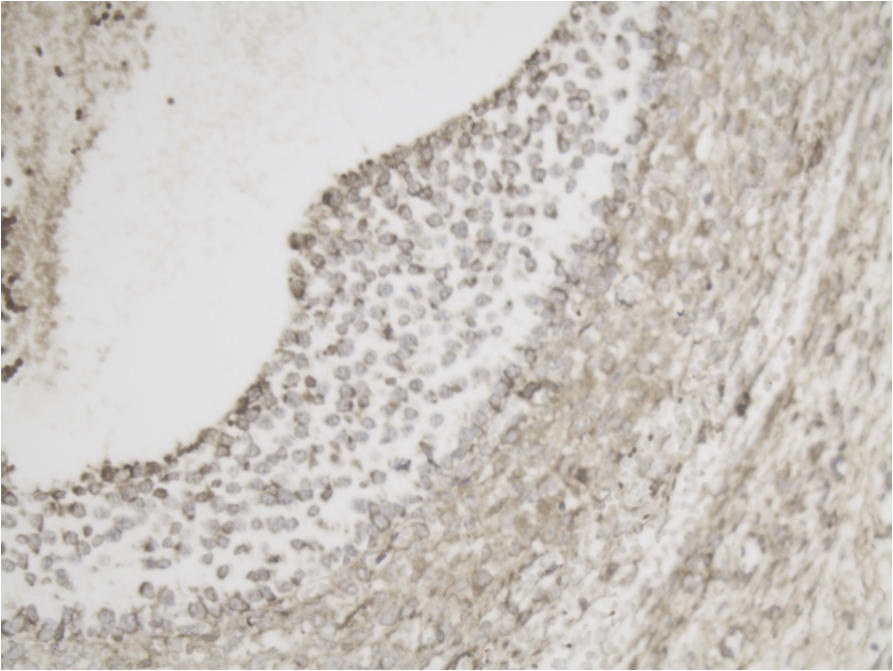
Representative slide of detail Tertiary follicle for MMP-2 10×20 magnification showing intense expression of MMP-2 especially at the inner layer of the follicle.

**Figure 9:**
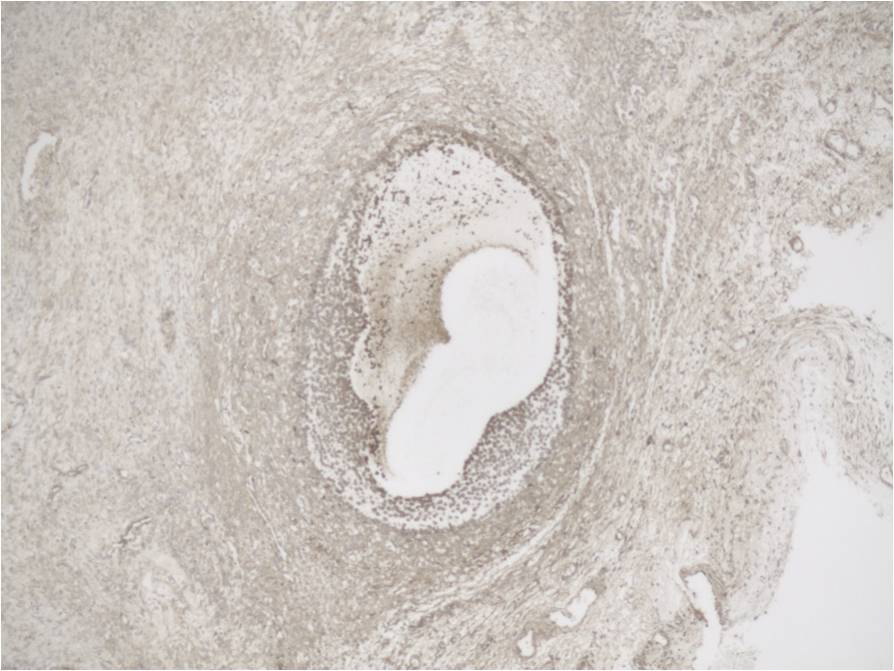
Representative slide of overview Tertiary follicle for MMP-14 5×10 magnification.

**Figure 10:**
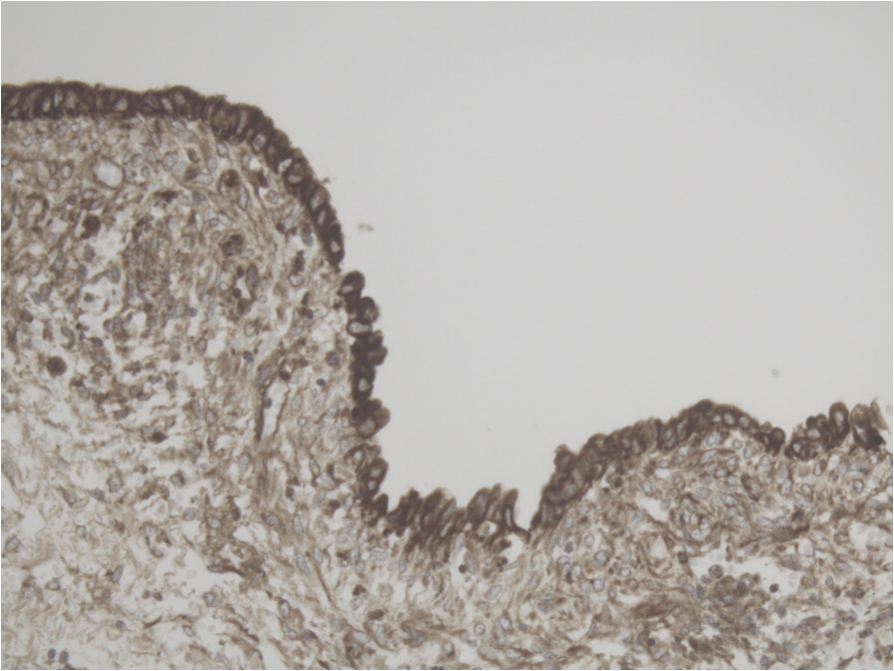
Representative slide of Corpus luteum for MMP-2 10×20 magnification with intense expression of MMP-2.

**Figure 11:**
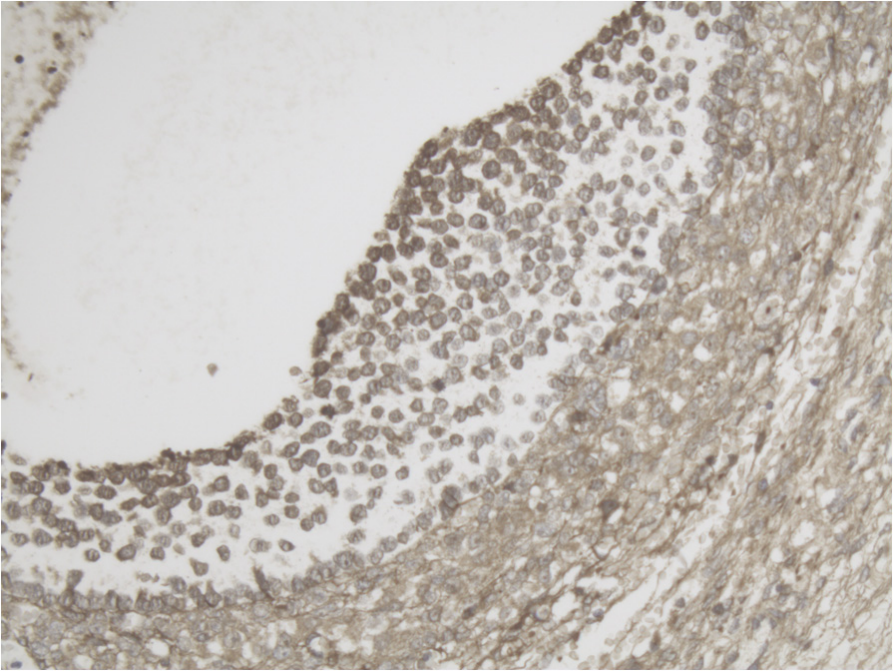
Representative slide of detail Tertiary follicle for MMP-14 10×20 magnification also showing intense expression at the inner layer of the follicle for MMP-14.

**Figure 12:**
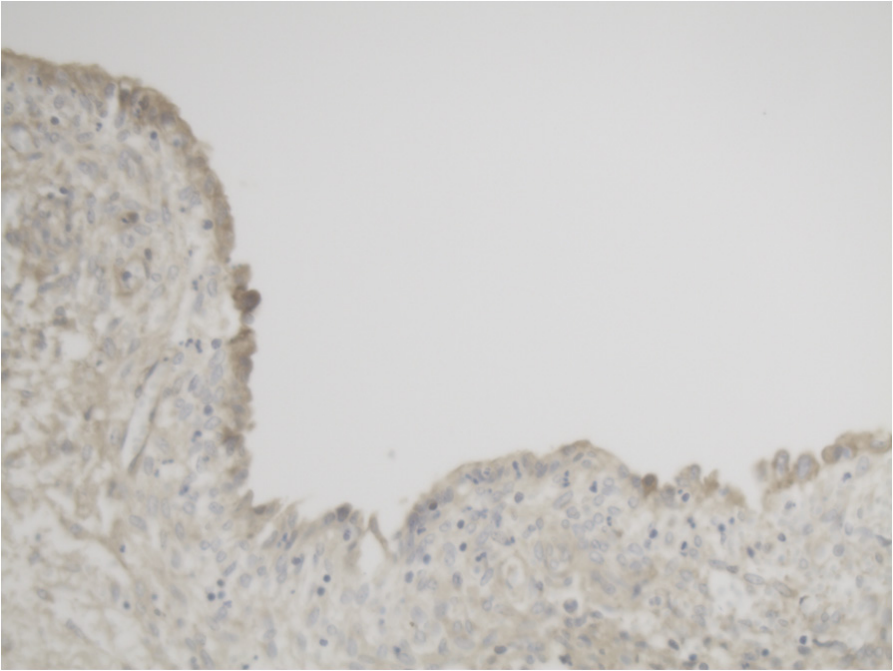
Representative slide of Corpus luteum for MMP-14 10×20 magnification with less intense expression of MMP-14.

In most primordial follicles, no MMP-14 protein is detected, but MMP-2 is present.

In one patient, some cells of some primordial follicles did express MMP-14 and some cells did not show MMP-14 expression.

Stromal staining for both MMP-14 and MMP-2 around primordial follicles was diffuse and weak and therefore not classified as positive.

In small primary/growing follicles the expression of both MMP-14 and MMP-2 is already more positive than in primordial follicles. The concentration of both proteins is highest at the inner layer of the granulosa cell layer next to the follicular fluid compartment.

High MMP-14 and MMP-2 expression in the inner layer of the granulosa cells is still present in one of the tertiary follicles we examined. In the other tertiary follicle the high expression at the inner layer of both proteins seemed less. We also observed high MMP-2 expression in the stromal cells of the ovary adjacent to the tertiary follicle, while little stromal MMP-14 expression was found.

In the corpus luteum MMP-2 is present with intense staining, especially in the inner layer of the granulosa cells, the outer layer had little expression of MMP-2. MMP-14 staining is less in both in the granulosa and theca cell layer.

The atretic follicle had diffuse MMP-14 and MMP-2 expression, which was not cellular. Since it did not have the typical pattern of cytoplasmatic staining for MMP-14 and pericellular staining for MMP-2, we did not classify it as positive. Ovarian surface epithelium does have expression of both proteins, which is the same in each slide, but in some specimens the staining was more intense than in others.

**Figure 13:**
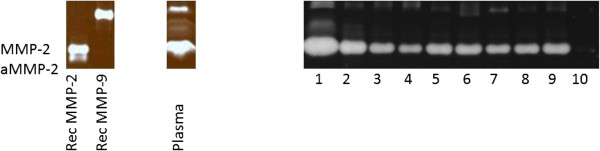
**Zymogram of follicular fluids at IVF.** Note that this figure was composed of different zymograms.

We collected follicular fluid of 9 patients and one cyst fluid of a benign cyst. IVF/ICSI characteristics of donors of follicular fluid are summarized in Table 3. Of these 10 patients zymographic evaluation of follicle fluid was performed. A typical example of a zymogram is depicted in Figure 13. In all patients pro-MMP-2 is present co-migrating with recombinant MMP-2. However, no separate band for activated MMP-2 was found in these zymograms. Patient 1 and patient 8 became pregnant, in patient 1 a high concentration of pro-MMP-2 was found. Patient 8 did not seem to differ from the other patients. The benign cystic fluid had very little MMP-2.

**Table 3:** IVF/ICSI characteristics of donors of follicular fluid used for zymography

**Age (years)**	**Indication**	**Primary/secundary infertility**	**Time infertility (years)**	**IVF/ICSI**	**Dose FSH**	**Amount follicular fluid**	**Number oocytes in tube**	**Total number oocytes**	**Pregnant**
28	Irregular cycle	Primary	7	IVF	225	41	2	5	+
30	PCOS	Primary	3	IVF	187.5	42	0	4	-
32	eci	Primary	3	IVF	225	36	3	8	-
40	Male factor	Secundary	2	ICSI	100	34	5	10	-
25	Male factor	Primary	1	ICSI	112.5	34	4	8	-
36	Male factor	Secundary	3	Half/half	150	34	6	10	-
34	Male factor	Primary	2	ICSI	150	30	5	14	-
31	Male factor	Primary	2	ICSI	112.5	25	2	5	+
37	Male factor	Secundary	1	ICSI	300	39	7	19	-
63	Benign cyst	n.a.				80	-	-	n.a.

IVF = In Vitro Fertilisation, ICSI = Intracytoplasmatic Sperm Injection, FSH = Follicle Stimulating Hormone, n.a. = not applicable, PCOS = PolyCystic Ovary Syndrome.

In the IVF patients also bands with gelatinase-activity with higher molecular weight were observed. They were less in density and may represent MMP-9.

## Discussion

The present study used immunohistochemistry to determine that MMP-14 and MMP-2 are present in various stages of human follicular development, which is consistent with the results of previous animal studies using FISH and zymography following gonadotrophic stimulation [6,16,17]. The present study also used zymography to demonstrate the presence of pro-MMP-2 in follicular fluid from IVF patients.

For both MMP-14 and MMP-2, a large proportion of the follicular and the ovarian surface epithelial cells were found to be positive, and the more advanced the development of the follicle, the greater the staining intensity (Table 2). Our findings in the corpora lutea appear to conflict with the findings of Manase et al. [12], though only two corpora lutea were examined in the present study and Manase et al. did not study follicles prior to ovulation. With respect to stromal staining, we found a different staining pattern for MMP-2 than the pattern observed by Lind et al. [11]. MMP-2 expression was found in stromal cells in the vicinity of the tertiary follicle and not around primordial follicles in the present study, while Lind et al. found stromal staining around the primordial follicles [11].

A difference was seen between the immunohistochemical staining patterns for MMP-14 and those for MMP-2: the former exhibit mostly cytoplasmatic and the latter mostly pericellular staining. The presence of MMP-2 in follicular fluid cannot be established by immunohistochemistry, though it has been demonstrated using zymography in animals, ELISA of pooled follicular fluids from IVF patients [8-10,18] and, as in the present study, zymography of such fluids.

Previously, in addition to immunohistochemistry and zymography, mRNA expression of MMP-14 and MMP-2 was used to study ovarian physiology, but only in the corpora lutea [12].

During its development, the human follicle increases in size by a factor 400 (illustrated by Figures 2-4 to 8 and 11). This requires extensive basement-membrane and extracellular matrix remodeling around the follicle, which continues during and after ovulation in the corpus luteum. Various studies indicate that MMP-14 plays a crucial role in this growth process; in particular, this is borne out by the phenomenon that MMP-14-deficient mice do not reproduce, whereas MMP-2-deficient mice do. Most of the present study’s findings show the presence of both MMP’s in follicular development. However, in all but one of the patients, no MMP-14 expression in primordial follicles was found but MMP-2 expression was observed; however, in a single patient – inexplicably – some primordial follicles exhibited MMP-14 and MMP-2 expression. This is inconsistent with the reported crucial role of MMP-14 in follicle growth. A MMP-2 stimulating pathway not involving MMP-14 has not been described in this context.

Strikingly, most MMP-14 and MMP-2 expression was found at the inner surface of the follicle, rather than at the follicular interface with the ovarian stroma. It may be reasonable to expect greater MMP expression at that interface, where remodeling of the extracellular matrix occurs. Interestingly, MMP-14 is known to respond not only to chemical but also physical stimuli [19,20]. It may be that the increasing pressure in the growing follicle stimulates MMP-14 expression and activity.

Two shortcomings of this study are its small sample size and its cross-sectional design. The latter meant that it was impossible to determine the relative ages of different primordial follicles in the same patient. An ideal study would follow a large number of follicles through their entire maturation process, but in practice this is impossible.

Our zymography results are comparable with those of other studies [8,12]. In the benign cystic fluid, little MMP-2 was found. MMP-2 is 72 kD in its inactive form and 66 kD following activation by MMP-14. The SDS in the zymography gels partly unfolds the inactive enzyme, rendering it active and detectable [21]. As zymography for MMP-2 is always performed under non-reducing conditions, the actual height of the bands is altered in most cases to 68 kD for the inactive form and 62 kD for the active form. In equine follicular fluid, also most of the MMP-2 that is detected by zymography is inactive [9]. In human corpora lutea extracts, the highest concentration of active MMP-2 is found in the late luteal phase [12]. In our IVF follicular fluids, we found no activated MMP-2, as was described by Lind et al. [11].

MMP-2 is detected in the follicular fluid, intracellularly in the follicle, and can be found extracellularly in the stroma, where it is probably recycled rapidly. A disadvantage of immunohistochemistry is that it only detects bound MMP-2. Furthermore, the monoclonal antibody used in this study probably does not detect differences between inactive and active MMP-2 [13].

## Conclusions

In conclusion, it has been demonstrated that MMP-14 and MMP-2 are expressed in developing human ovarian follicles and that there is MMP-2 present in the follicular fluid. These results are consistent with the patterns revealed by mRNA expression and zymography in numerous animal studies and a few human studies.

## Competing interests

No competing interest by any of the authors.

## Authors’ contributions

MCV designed the study, scored the immunoassays, performed zymography and drafted the manuscript. JL carried out and scored the immunoassays under supervision of EdB. AW conceived of the study, supervised scoring the immunoassays and helped to draft the manuscript. JS contributed to the design of the study and provided IVF-data. AK and LM supervise the study and helped to draft the manuscript. All authors read and approved the final manuscript.
